# A Comprehensive Analysis of Dermatological Manifestations in Lower Limb Para-Athletes

**DOI:** 10.26502/acmcr.96550745

**Published:** 2026-01-30

**Authors:** Andre Aabedi, Vera Wang, Devendra K. Agrawal

**Affiliations:** Department of Translational Research, College of Osteopathic Medicine of the Pacific, Western University of Health Sciences, Pomona, California 91766, USA

**Keywords:** Dermatitis, Friction and shear forces, Lower limb amputees, Pressure ulcers, Prosthetic skin complications, Prosthetic socket interface

## Abstract

In this article, a critical evaluation of current evidence is presented on the prevalence, mechanisms, prevention, and management of sport-related skin complications in amputee athletes using lower limb prostheses, highlighting emerging technologies aimed at improving skin health and athletic performance. Skin complications were highly prevalent, affecting 34%-77% of lower limb prosthesis users, with even greater frequency among athletes. The most common conditions included maceration, friction blisters, pressure ulcers, contact dermatitis, verrucous and epidermal hyperplasia, and bacterial or fungal infections. Key risk factors encompassed poor prosthetic fit, elevated activity levels, increased perspiration, prolonged wear, inadequate hygiene, and hot or humid environments. Mechanical loading and shear stress at the socket–skin interface, compounded by moisture and heat retention, were central pathophysiologic drivers. Prevention and management strategies emphasize meticulous prosthetic fitting, consistent hygiene practices, routine skin inspection, and prompt intervention for early lesions. Multidisciplinary collaboration among dermatologists, prosthetists, and rehabilitation specialists improves detection and management outcomes. Recent innovations including vented liners, temperature-regulating materials, antimicrobial coatings, and sensor-based “smart prosthetics” show promise in reducing friction, heat, and infection risk, though evidence in athletic populations remains limited. Cutaneous complications in amputee athletes are common, multifactorial, and preventable. Optimal prosthetic fit, athlete education, and coordinated interdisciplinary care are essential for minimizing morbidity and maintaining athletic participation. Technological advancements such as real-time pressure and temperature monitoring, antimicrobial materials, and adaptive socket systems may transform prevention strategies, but further sport-specific, longitudinal research is required to validate their clinical impact and guide evidence-based practice.

## 1. Introduction

Skin complications represent a significant challenge for amputee athletes using lower limb prostheses, arising from the distinctive mechanical and environmental stresses imposed during athletic activity. The prosthetic socket-skin interface experiences repetitive friction, shear forces, and elevated humidity levels that collectively predispose athletes to various dermatologic conditions [1]. These complications can interrupt prosthesis use, compromise athletic performance, and substantially diminish quality of life [2].

Common cutaneous manifestations include maceration, friction blisters, pressure ulcers, allergic contact dermatitis, epidermal hyperplasia, hyperhidrosis, and bacterial or fungal infections [3]. The prevalence of skin problems among lower limb amputees is notably high—studies report rates approaching 70% in general amputee populations—and may be elevated further in athletes due to heightened activity levels and perspiration [3]. Ill-fitting prostheses, suboptimal socket design, and prolonged wear during sports substantially amplify these risks [4]. Allergic reactions to prosthetic materials and liners have also been documented, with patch testing identifying causative allergens in a considerable proportion of affected individuals.

Sport-specific prostheses, though engineered to enhance performance, may paradoxically introduce additional cutaneous risks when improperly fitted or inadequately maintained. Effective prevention hinges on meticulous skin care protocols, routine residual limb inspection, optimal prosthetic fitting, and timely management of incipient skin changes [5]. Multidisciplinary care integrating dermatology, rehabilitation medicine, and prosthetists is critical for early detection and intervention, thereby minimizing complications and sustaining athletic participation. Despite the high prevalence and clinical impact of these complications, additional research is warranted to characterize sport-specific risk factors and establish evidence-based prevention strategies for this population [6]. This critical review is a comprehensive analysis of the types, causes, prevention, and management of sport-related skin complications in lower-limb prosthesis users.

## 2. Method

A comprehensive literature search was conducted across PubMed, Scopus, Embase, and SPORTDiscus databases using combinations of the terms “lower limb amputation,” “prosthesis,” “athlete,” “sports,” and “skin complications.” Peer-reviewed studies published in English within the past two decades were included if they examined cutaneous outcomes in lower limb prosthesis users engaged in athletic or high-activity contexts. Data were extracted on epidemiology, risk factors, complication types, preventive strategies, and technological innovations.

## 3. Overview of Skin Biomechanics in Prosthesis Use

The residual limb skin of amputee athletes using prostheses experiences distinctive mechanical loading patterns—including pressure, shear, and friction—that can fundamentally alter normal skin physiology and trigger cutaneous complications. Mechanical loading induces deformation of skin and underlying soft tissues, initiating cellular and microvascular changes with clinically significant consequences. Sustained or repetitive pressure and shear forces at the prosthetic socket interface compromise local perfusion, precipitating ischemia and elevating the risk of ulceration and tissue necrosis [7]. Shear stresses are particularly implicated in both deep tissue injury and superficial lesions, as they distort dermal and subcutaneous architecture, disrupting microcirculation and promoting inflammatory cascades [8].

**Figure 1:**
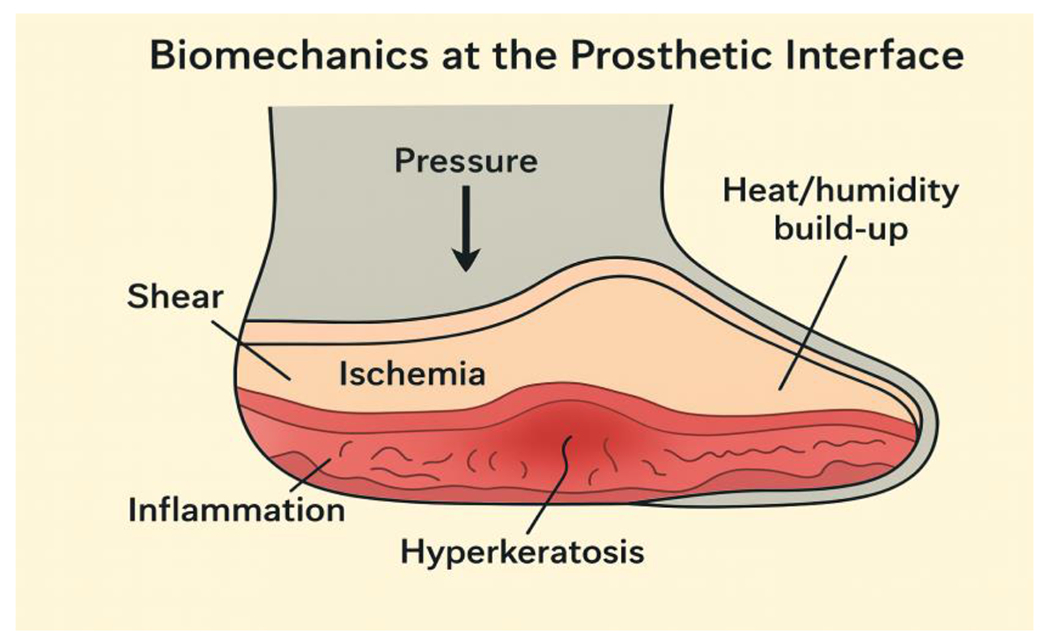
The schematic diagram illustrates the biomechanical forces—pressure, shear, and friction—acting at the skin–socket interface.

At the cellular level, mechanical forces activate mechanotransduction pathways in keratinocytes, fibroblasts, and other resident skin cells, triggering dysregulated cytokine release, sustained inflammation, and compromised regenerative capacity. Chronic mechanical stress can induce compensatory hyperkeratosis and epidermal hyperplasia, with severe cases progressing to cellular necrosis or aberrant differentiation [9]. The skin’s biomechanical response demonstrates considerable heterogeneity, modulated by individual factors including baseline vascular integrity, pre-existing skin condition, and the local microenvironment—particularly humidity and temperature gradients within the prosthetic socket (Figure 1) [9].

For amputee athletes, elevated activity levels and repetitive cyclical loading during sport impose amplified physiological stress, substantially increasing vulnerability to dermatologic sequelae including contact irritation, pressure ulcers, and verrucous hyperplasia [10]. Systematic monitoring and mitigation of these mechanical forces are therefore essential to preserving skin integrity and optimizing prosthetic function in this population [11].

Skin complications in amputee athletes are heavily influenced by how prosthetic liners, socket materials, sweat, and heat interact during activity. Materials with poor ventilation and heat retention trap moisture against the skin, increasing sweat production and causing maceration—which heightens the risk of irritation, rashes, and ulcers [12]. Silicone liners, though cushioning, tend to promote sweating and require daily cleaning to prevent hygiene-related problems [13]. Exercise significantly raises temperature and humidity inside the socket, intensifying sweat production and discomfort. Research using sensors and thermal imaging confirms that skin temperature and moisture levels spike after physical activity, creating hot spots and sweat accumulation that can damage skin integrity-especially during intense or prolonged exercise.

Newer technologies show promise in addressing these issues. Vented liner-socket systems effectively reduce humidity and perceived sweating while maintaining secure fit [12]. Similarly, phase-change material (PCM) liners and sockets with cooling channels help regulate skin temperature and improve comfort, potentially preventing skin breakdown [14]. However, more research is needed to confirm whether these innovations provide lasting benefits for athletic populations [14].

## 4. Epidemiology and Risk Factors

Skin problems are common and clinically important in lower limb amputees who use prostheses, particularly among athletes. Studies report that 34% to 77% of prosthesis users experience cutaneous complications, with variation depending on the population studied and assessment methods used [15]. General amputee populations show skin problem rates of 41% to 74%, with higher frequencies among more active individuals and those wearing prostheses for extended daily periods [16]. A large Dutch survey found that 63% of users reported skin issues within the past month, with younger, more active amputees at greatest risk [17]. Another recent study reported a 77% one-month prevalence, though severe problems were not linked to higher BMI [18].

Athletes face elevated risk due to increased mechanical stress, repetitive friction, and prolonged prosthesis use during training and competition [19]. Athletic activity and employment status are independent risk factors for skin complications, with active amputees showing higher rates of dermatologic problems. Common sport-related issues include friction blisters, pressure ulcers, contact dermatitis (both irritant and allergic), epidermal hyperplasia, and bacterial or fungal infections [16]. These complications significantly affect daily life, often reducing prosthesis use, limiting athletic participation, and diminishing quality of life [2]. Fungal and bacterial infections, intertriginous dermatitis, and eczema occur frequently, especially with increased humidity and prolonged wear. Additional risk factors include summer season and daily prosthesis use exceeding eight hours [20].

Key risk factors for sport-related skin complications in lower limb amputee athletes include prosthetic fit, amputation level, activity intensity, hygiene practices, and environmental conditions. Prosthetic fit is the primary determinant of skin health. Poor fit generates friction, pressure, and shear forces that cause irritant and allergic contact dermatitis, ulcers, and epidermal hyperplasia. Frequent adjustments and individualized fitting are essential, particularly for highly active athletes [21]. While sport-specific prostheses reduce risk, they do not eliminate it [4].

Amputation level significantly influences complication rates. Proximal amputations create greater mechanical stress and biomechanical demands than distal amputations, increasing vulnerability to skin problems [22]. Higher-level amputations may also impair thermoregulation, further elevating risk during intense activity [19]. Activity intensity directly correlates with skin complications. Athletes in high-impact or endurance sports face elevated risk from repetitive loading, prolonged prosthesis use, and sustained exposure to friction and moisture [15]. Hygiene practices play a critical role. Both excessive washing with antibacterial soaps and inadequate stump or liner hygiene increase susceptibility to infections and dermatitis. Smoking compounds this risk [17]. Optimal prevention involves regular but moderate cleaning, prompt treatment of skin breaks, and avoidance of irritants, as emphasized by American College of Sports Medicine guidelines [23]. Environmental factors including heat, humidity, and facility cleanliness further modulate risk. Elevated temperature and humidity increase perspiration and moisture accumulation, promoting maceration and infection. Contaminated equipment and poor facility hygiene present additional hazards [2].

## 5. Types of Cutaneous Complications

Lower limb amputee athletes face elevated risk for skin complications during sports due to increased mechanical stress, friction, and occlusion at the skin-prosthesis interface. These complications primarily fall into two categories: mechanical injuries and dermatitis. Mechanical injuries include friction blisters, abrasions, pressure ulcers, calluses, and verrucous hyperplasia, arising from repetitive shear forces, pressure points, and ill-fitting sockets during high-intensity or prolonged activity. Ulcers and irritations are most frequently documented, along with calluses and inclusion cysts [10]. Athletes experience particularly high rates of blisters and abrasions due to increased loading and movement, which can lead to secondary infection [19].

Dermatitis manifests as either irritant or allergic contact dermatitis. Irritant dermatitis results from physical and chemical irritation—sweat, heat, and friction—while allergic contact dermatitis stems from sensitization to prosthetic materials or liners. Allergic reactions account for a substantial proportion of residual limb dermatitis cases, warranting patch testing for persistent or atypical presentations [16]. Both types present with erythema, pruritus, and scaling, making clinical differentiation challenging [21]. Bacterial and fungal infections represent the primary infectious complications in amputee athletes. Bacterial infections are more prevalent, typically manifesting as cellulitis or localized abscesses at the residual limb-prosthesis interface. Contributing factors include elevated humidity, friction, and microtrauma from prosthetic use [3]. Fungal infections, though less frequent, are predominantly caused by dermatophytes thriving in the moist prosthetic socket environment [16]. Prolonged athletic activity, excessive perspiration, and inadequate hygiene practices amplify risk for both infection types [19].

**Figure 2:**
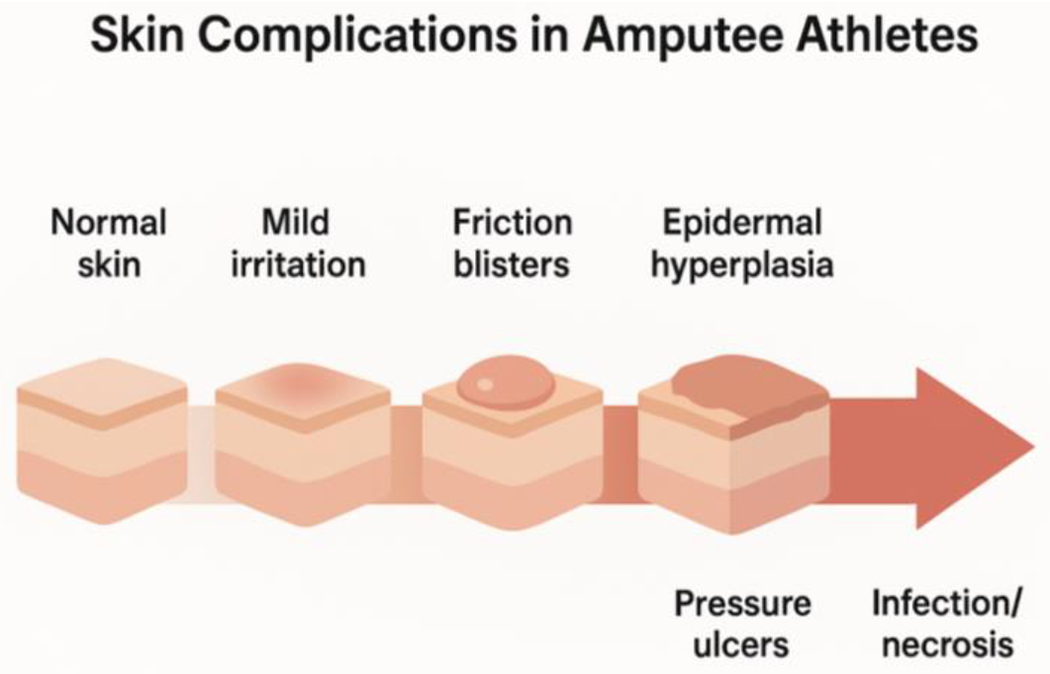
The digarma demonstrates progression of skin injury in amputee athletes and where interventions can act.

Chronic skin alterations result from repetitive mechanical stress, sustained moisture exposure, and allergic reactions. Common manifestations include irritant and allergic contact dermatitis, verrucous hyperplasia, epidermal hyperplasia, calluses, inclusion cysts, and chronic ulcerations [2]. Allergic contact dermatitis frequently relates to prosthetic materials or liners, with patch testing confirming sensitization in a substantial proportion of affected athletes. Persistent friction and pressure produce hyperkeratotic lesions, cyst formation, and ulceration that can significantly impair prosthesis tolerance and athletic performance [10]. Athletes also commonly report excessive perspiration and cold skin, reflecting altered thermoregulation and increased physiological demands (Figure 2) [17]. Early recognition and coordinated multidisciplinary management are critical to preserving prosthetic function and maintaining athlete quality of life.

## 6. Prevention and Management

Skin complications affect 36% to 70% of lower limb amputee athletes using prostheses, representing a significant clinical challenge [2]. Common disorders include ulcers, irritations, calluses, verrucous hyperplasia, and contact dermatitis, arising from mechanical shear, friction, elevated humidity, and immune vulnerability at the residual limb [24]. These complications frequently reduce prosthesis tolerance, compromise athletic performance, and diminish quality of life [3].

Optimal prosthetic fit and rigorous hygiene form the foundation of prevention. Technological advances, including microprocessor-controlled variable-stiffness ankle-foot devices, demonstrate improved biomechanics and may reduce abnormal loading patterns that contribute to skin breakdown [25]. Sport-specific prostheses are engineered to accommodate athletic demands while minimizing injury risk [4]. Regular evaluation of prosthetic fit, socket interface, and liner materials is essential, as improper fit and inappropriate materials are primary contributors to cutaneous problems [19].

Skin care protocols should emphasize daily washing and thorough drying of the residual limb while avoiding antibacterial soaps that may provoke irritation.[26] The American College of Sports Medicine recommends routine surveillance for skin infections, strict hygiene adherence, and prompt exclusion from sport until lesions resolve [27]. Multidisciplinary collaboration—integrating dermatology, rehabilitation medicine, and prosthetics expertise-enables early detection and intervention critical for sustaining athletic participation [27,28].

Athletes should be trained in self-examination techniques using mirrors and encouraged to engage family members or support persons in routine skin monitoring, particularly when visual limitations exist. Education must address modifiable risk factors including smoking cessation and elimination of unnecessary antibacterial products [17]. Athletes require individualized guidance addressing the unique demands of their sport—including intensified mechanical loading, increased perspiration, and environmental exposures—along with targeted risk mitigation strategies [29].

## 7. Future Directions

Emerging strategies to mitigate sport-related cutaneous complications in lower limb amputee athletes focus on three pivotal technological innovations: smart prosthetics, antimicrobial materials, and telemonitoring systems [30]. Next-generation prosthetics integrate real-time sensor technologies to continuously monitor pressure distribution, temperature fluctuations, and tissue health at the residual limb-socket interface [31]. Wireless, battery-free sensors transmit data to external devices, enabling early detection of impending skin breakdown and dynamic socket optimization—critical capabilities for preventing friction injuries and pressure ulcers in athletes [32]. Emerging neuroprosthetic feedback systems aim to restore sensory perception and reduce cognitive load during prosthesis use, potentially lowering complication risk through improved gait mechanics and enhanced prosthetic embodiment [33,34].

Advanced antimicrobial coatings for prosthetic sockets and liners address the elevated infection risk associated with prolonged skin contact and perspiration during athletic activity [35]. Surface technologies incorporating metal and metal oxide nanoparticles and phytochemical compounds demonstrate broad-spectrum antimicrobial efficacy, biofilm disruption, and superior biocompatibility, with potential to substantially reduce infection rates and improve tissue integration [36,37]. These innovations are particularly salient given rising antimicrobial resistance in athletic populations [38].

Remote monitoring platforms utilizing wearable sensors and mobile health technologies enable continuous tracking of skin integrity, prosthetic fit, and activity patterns. End-users and clinicians emphasize the importance of lightweight, unobtrusive systems that generate actionable insights, facilitating timely intervention and individualized prosthetic management [39]. These platforms may additionally support clinical reimbursement and decision-making through objective documentation of complications and prosthetic performance [30,40].

These converging innovations hold promise for reducing the incidence and severity of cutaneous complications, enhancing athlete safety, and improving long-term outcomes in this population. Future research should focus on sport-specific studies and standardized reporting.

## 8. Conclusion

Skin complications remain a prevalent and consequential challenge for amputee athletes using lower limb prostheses, arising from the complex interplay of mechanical loading, moisture accumulation, and material interactions at the socket–skin interface. These dermatologic issues not only compromise comfort and prosthesis tolerance but also limit athletic participation and performance. Preventive strategies centered on optimal prosthetic fit, routine skin surveillance, strict hygiene, and early multidisciplinary management are critical to sustaining sport participation and minimizing morbidity. Ongoing advances in prosthetic design—such as temperature-regulating liners, antimicrobial coatings, and sensor-based feedback systems—offer promising avenues for real-time monitoring and risk reduction. However, the current literature remains limited by small sample sizes, heterogeneous methodologies, and a lack of sport-specific data. Future research should prioritize prospective studies that define the biomechanical and environmental determinants of skin health in athletic contexts, evaluate the clinical utility of emerging technologies, and establish standardized, evidence-based guidelines for prevention and management. By integrating innovation with individualized care, clinicians and researchers can substantially improve outcomes and quality of life for amputee athletes striving for high-level performance.

## Key Points

- Skin complications are highly prevalent among lower limb amputee athletes, with rates up to 70%, and significantly impact prosthesis use, athletic performance, and quality of life.
- Mechanical forces at the prosthetic socket—pressure, shear, and friction—trigger cellular and microvascular changes, contributing to ulcers, blisters, and epidermal hyperplasia.
- Sweat, heat, and occlusion within the prosthetic socket exacerbate skin maceration, friction injuries, and infections.
- Risk factors include prosthetic fit, amputation level, activity intensity, hygiene practices, and environmental conditions.
- Cutaneous complications encompass mechanical injuries (blisters, abrasions, pressure ulcers, calluses) and dermatitis (irritant or allergic), along with bacterial and fungal infections.
- Prevention strategies emphasize optimal prosthetic fit, daily hygiene, routine skin inspection, and individualized adjustments, particularly for highly active athletes.
- Multidisciplinary management involving dermatology, rehabilitation, and prosthetics specialists is critical for early detection and intervention.
- Emerging technologies—vented or temperature-regulating liners, antimicrobial coatings, smart prosthetic sensors, and telemonitoring—show promise for reducing skin complications in athletes.
- Education and training for athletes on self-monitoring, risk factor modification, and proper prosthetic maintenance are essential components of prevention.
- Future research should focus on sport-specific studies, standardized reporting, and evaluation of innovative prosthetic technologies to improve skin health outcomes.

## References

[1] GBD 2021 US Obesity Forecasting Collaborators. National-level and State-level Prevalence of Overweight and Obesity Among Children, Adolescents, and Adults in the USA, 1990-2021, and Forecasts up to 2050. The Lancet 404 (2024): 2278–2298. 10.1016/S0140-6736(24)01548-4 PMC11694015 39551059

[2] Phil McEwan, Faurby M, Lübker C, The Evolving Burden of Obesity in the U.S.: A Novel Population-Level System Dynamics Approach. Journal of Medical Economics 28 (2025): 1512–1525. 40880254 10.1080/13696998.2025.2554518

[3] Qiyuan K L Mechanisms of Action and Therapeutic Applications of GLP-1 and Dual GIP/GLP-1 Receptor Agonists. Front Endocrinol 15 (2024): 1431292. 10.3389/fendo.2024.1431292 PMC11304055 39114288

[4] Pillarisetti L, Agrawal DK. Semaglutide: Double-edged Sword with Risks and Benefits. Arch Intern Med Res 8 (2025): 1–13. 39902055 10.26502/aimr.0189 PMC11790292

[5] Nauck Michael A, Müller Timo D. Incretin Hormones and Type 2 Diabetes. Diabetologia 66 (2023): 1780–1795. 37430117 10.1007/s00125-023-05956-x PMC10474001

[6] Zafer M, Tavaglione F, Manuel RG, GLP-1 Receptor Agonists and Glucagon/GIP in Obesity Management: Review Article. Aliment Pharmacol Ther 61 (2025): 1872–1888. 40364529 10.1111/apt.70196 PMC12323726

[7] Nauck MA, Daniel RQ, Jakob W, The Evolving Story of Incretins (GIP and GLP-1) in Metabolic and Cardiovascular Disease: A Pathophysiological Update. Diabetes Obes Metab 23 (2021): 5–29. 34310013 10.1111/dom.14496

[8] Holst JJ. The Physiology of Glucagon-Like Peptide 1. Physiol Rev 87 (2007): 1409–1439. 17928588 10.1152/physrev.00034.2006

[9] Seufert J, Gallwitz B. The Extra-Pancreatic Effects of GLP-1 Receptor Agonists. Diabetes Obes Metab 16 (2014): 673–688. 24373150 10.1111/dom.12251

[10] Mouawad M, Nabipur L, Agrawal DK. Impact of Antidepressants on Weight Gain: Underlying Mechanisms and Mitigation Strategies. Arch Clin Biomed Res 9 (2025): 183–195. 40444017 PMC12121960

[11] Karakasis P, Patoulias D, Theofilis P, GLP-1 Receptor Agonists and Myocardial Perfusion. Int J Mol Sci 13 (2025): e15023. 10.3390/ijms26073050 PMC11988964 40243679

[12] Giugliano D, Scappaticcio L, Longo M, GLP-1 Receptor Agonists and Cardiorenal Outcomes in Type 2 Diabetes: An Updated Meta-Analysis of Eight CVOTs. Cardiovasc Diabetol 20 (2021): 189. 34526024 10.1186/s12933-021-01366-8 PMC8442438

[13] Ludwig MQ, Todorov PV, Egerod KL, Single-Cell Mapping of GLP-1 and GIP Receptor Expression Supports Functional Heterogeneity. Diabetes 70 (2021): 1945–1958. 34176785 10.2337/dbi21-0003 PMC8576419

[14] Ilias P, Tsioufis K, Katsi V. Spotlight on the Mechanism of Action of Semaglutide. Curr Issues Mol Biol 46 (2024): 14514–14541. 39728000 10.3390/cimb46120872 PMC11674233

[15] Marso SP, Bain SC, Consoli A, Semaglutide and Cardiovascular Outcomes in Patients with Type 2 Diabetes. N Engl J Med 375 (2016): 1834–1844. 27633186 10.1056/NEJMoa1607141

[16] Kommu S, Philip W. Semaglutide. StatPearls. StatPearls Publishing (2025).

[17] Fornes A, Huff J, Pritchard RL, Once-Weekly Semaglutide for Weight Management: A Clinical Review. J Pharm Technol 38 (2022): 239–246. 35832567 10.1177/87551225221092681 PMC9272494

[18] Rubino D, Abrahamsson N, Davies M, Effect of Continued Weekly Subcutaneous Semaglutide vs Placebo on Body Weight in Adults with Overweight or Obesity - The STEP 4 Randomized Clinical Trial. JAMA 325 (2021): 1414–1425. 33755728 10.1001/jama.2021.3224 PMC7988425

[19] Wilkinson L, Thomas HH, Peter NL, Effect of Semaglutide 2.4 mg Once Weekly on 10-Year Type 2 Diabetes Risk in People with Overweight or Obesity. Obesity 31 (2023): 2249–2259. 37605636 10.1002/oby.23842

[20] Alireza H, Aayushi S, Jahangir K, Glucagon-Like Peptide-1 Receptor Agonists and Major Adverse Cardiovascular Events in Patients with and Without Diabetes: A Meta-Analysis of Rawithized-Controlled Trials. Clin Cardiol 47 (2024): e24314. 38953365 10.1002/clc.24314 PMC11217813

[21] Ryan DH, Lingvay I, Helen MC, Semaglutide Effects on Cardiovascular Outcomes in People with Overweight or Obesity (SELECT): Rationale and Design. Am Heart J 229 (2020): 61–69. 32916609 10.1016/j.ahj.2020.07.008

[22] Patel S, Patel H, Mukadam S, Apolipoprotein B in the Risk Assessment, Diagnosis, and Treatment of Cardiometabolic Diseases. Cardiol Cardiovasc Med 9 (2025): 427–438. 41346943 10.26502/fccm.92920466 PMC12674602

[23] Ania MJ, Louis JA, Nadia NA, Tirzepatide Once Weekly for the Treatment of Obesity. N Engl J Med 387 (2022): 205–216. 35658024 10.1056/NEJMoa2206038

[24] Aronne LJ, Deborah BH, Carel W l R, Tirzepatide as Compared with Semaglutide for Obesity Treatment. N Engl J Med 393 (2025): 1654–1665. 40353578 10.1056/NEJMoa2416394

[25] Sardar MB, Zain AN, Muhammad B, Tirzepatide: A Novel Cardiovascular Protective Agent in Type 2 Diabetes Mellitus and Obesity. Curr Probl Cardiol 49 (2024): 102489. 38417475 10.1016/j.cpcardiol.2024.102489

[26] Song Z, Tang Y, Peng M, Pharmacogenomics of Tirzepatide: Genomic Insights into Dual GIP/GLP-1 Agonist Response in Type 2 Diabetes and Atherosclerosis. Pharmaceuticals 18 (2025): 1261. 41011133 10.3390/ph18091261 PMC12473131

[27] Smits Mark M, Daniël H. Van Raalte. Safety of Semaglutide. Front Endocrinol 12 (2021): 645563. 10.3389/fendo.2021.645563 PMC8294388 34305810

[28] Xin Z, Wang M, Wen Z, GLP-1 Receptor Agonists: Beyond Their Pancreatic Effects. Front Endocrinol 12 (2021): 721135. 10.3389/fendo.2021.721135 PMC8419463 34497589

[29] Chaudhry A, Gabriel B, Noor J, Tendency of Semaglutide to Induce Gastroparesis: A Case Report. Cureus 16 (2024): e10874596. 10.7759/cureus.52564 PMC10874596 38371020

[30] Smits Mark M, Daniël H. Van Raalte. Safety of Semaglutide. Front Endocrinol 12 (2021): 645563. 10.3389/fendo.2021.645563 PMC8294388 34305810

[31] Ghusn W, Maria DH, Glucagon-like Receptor-1 Agonists for Obesity: Weight Loss Outcomes, Tolerability, Side Effects, and Risks. Obes Pillars 12 (2024): 100127. 39286601 10.1016/j.obpill.2024.100127 PMC11404059

[32] Hathaway JT, Shah MP, Hathaway DB, Risk of Non-arteritic Anterior Ischemic Optic Neuropathy in Patients Prescribed Semaglutide. JAMA Ophthalmol 142 (2024): 732–739. 38958939 10.1001/jamaophthalmol.2024.2296 PMC11223051

[33] Smits, Mark M, Daniël H. Van Raalte. Safety of Semaglutide. Front Endocrinol 12 (2021): 645563. 10.3389/fendo.2021.645563 PMC8294388 34305810

[34] Nauck MA, Daniel RQ, Jakob W, The Evolving Story of Incretins (GIP and GLP-1) in Metabolic and Cardiovascular Disease: A Pathophysiological Update. Diabetes Obes Metab 23 (2021): 5–29. 34310013 10.1111/dom.14496

[35] Hiren P, Khunti K, Rodbard HW, Gastrointestinal Adverse Events and Weight Reduction in People with Type 2 Diabetes Treated with Tirzepatide in the SURPASS Clinical Trials. Diabetes Obes Metab 26 (2024): 473–481. 37853960 10.1111/dom.15333

[36] Rachel S, Papamargaritis D, Jack AS, Efficacy and Safety of Tirzepatide in Type 2 Diabetes and Obesity Management. J Obes Metab Syndr 32 (2023): 25–45. 36750526 10.7570/jomes22067 PMC10088547

[37] Jie G, Gao F, Jiang K, Risk of Biliary Diseases in Patients with Type 2 Diabetes or Obesity Treated with Tirzepatide: A Meta-analysis. J Diabetes Investig 16 (2025): 83–92. 10.1111/jdi.14340 PMC11693576 39569606

[38] Kamrul-Hasan ABM, Sunetra M, Deep D, Pancreatic Safety of Tirzepatide and Its Effects on Islet Cell Function: A Systematic Review and Meta-Analysis. Obes Sci Pract 10 (2024): e70032. 39720158 10.1002/osp4.70032 PMC11667760

[39] Safwan M, Mariam SB, Shahad AA, Gastrointestinal Safety of Semaglutide and Tirzepatide vs. Placebo in Obese Individuals without Diabetes: A Systematic Review and Meta-Analysis. Ann Saudi Med 45 (2025): 129–143. 40189856 10.5144/0256-4947.2025.129 PMC12542916

[40] Kamrul-Hasan ABM, Muhammad SA, Deep D, Tirzepatide and Cancer Risk in Individuals with and without Diabetes: A Systematic Review and Meta-Analysis. Endocrinol Metab 40 (2025): 112–124. 10.3803/EnM.2024.2164 PMC11898313 39814031

[41] Rahul M, Rishi R, Elshimy G, Adverse Events Related to Tirzepatide. J Endocr Soc 7 (2023): 016. 10.1210/jendso/bvad016 PMC9915969 36789109

[42] Shi FH, Hao L, Cui M, Efficacy and Safety of Once-Weekly Semaglutide for the Treatment of Type 2 Diabetes: A Systematic Review and Meta-Analysis of Randomized Controlled Trials. Front Pharmacol 9 (2018): 576. 29915538 10.3389/fphar.2018.00576 PMC5994433

[43] Munawar N, Mahato A, Rawat A, Tirzepatide Versus Semaglutide for Weight Loss in Overweight and Obese Adults: A Systematic Review and Meta-Analysis of Direct Comparative Studies. Cureus 17 (2025): e86080. 40666599 10.7759/cureus.86080 PMC12263181

[44] Kasagga A, Delvy R, Tooba H, Comparative Efficacy and Tolerability of Tirzepatide Versus Semaglutide at Varying Doses for Weight Loss in Non-diabetic Adults with Obesity: A Network Meta-Analysis of Randomized Controlled Trials. Cureus 17 (2025): e90335. 40978842 10.7759/cureus.90335 PMC12444735

[45] Dai H, Li Y, Lee YA, GLP-1 Receptor Agonists and Cancer Risk in Adults with Obesity. JAMA Oncol 11 (2025): 1186–1193. 40839273 10.1001/jamaoncol.2025.2681 PMC12371547

[46] Lamabadusuriya DA, Jayasena H, Bopitiya AK, Obesity-Driven Inflammation and Cancer Risk: A Comprehensive Review. Semin Cancer Biol 114 (2025): 256–266. 40714142 10.1016/j.semcancer.2025.07.007

[47] Deng T, Lyon CJ, Bergin S, Obesity, Inflammation, and Cancer. Annu Rev Path 11 (2016): 421–449. 27193454 10.1146/annurev-pathol-012615-044359

[48] Benjamin DJ, Daniel D Von Hoff. The Emerging Role of GLP-1 Receptor Agonists in Treating or Preventing Cancer. Cancer Drug Resist 7 (2024): 49. 39931650 10.20517/cdr.2024.116 PMC11810457

[49] Bera S, Lisa CG, Brynne D, GLP1 Receptor Agonism Alters Growth and Therapeutic Response in Prostate Cancer. Endocr Relat Cancer (2025): E250185. 41257454 10.1530/ERC-25-0185 PMC12713343

[50] Basile C, Merolla A, Mancusi C, Effect of Incretin-Based Therapies on Blood Pressure: A Systematic Review and Meta-Analysis. Eur J Prev Cardiol (2025): zwaf560. 40899050 10.1093/eurjpc/zwaf560

[51] Ding L, Jin Z. Glucagon-like Peptide-1 Activates Endothelial Nitric Oxide Synthase in Human Umbilical Vein Endothelial Cells. Acta Pharmacol Sin 33 (2012): 75–81. 22120969 10.1038/aps.2011.149 PMC4010269

[52] Crajoinas RO, Oricchio FT, Pessoa TD, Mechanisms Mediating the Diuretic and Natriuretic Actions of the Incretin Hormone Glucagon-like Peptide-1. Am J Physiol 301 (2011): F355–F363. 10.1152/ajprenal.00729.2010 21593184

[53] Hong CT, Chen JH, Hu CJ, Role of Glucagon-like Peptide-1 Receptor Agonists in Alzheimer’s Disease and Parkinson’s Disease. J Biomed Sci 31 (2024): 102. 39501255 10.1186/s12929-024-01090-x PMC11539687

[54] Glotfelty EJ, Olson L, Karlsson TE, Glucagon-like peptide-1-based receptor agonists as a treatment for Parkinson’s disease. Cells & Systems (2020). 10.1080/13543784.2020.1764534 PMC10477949 32412796

[55] Kupnicka P, Małgorzata K, Justyna Ż, GLP-1 Receptor Agonists: A Promising Therapy for Modern Lifestyle Diseases with Unforeseen Challenges. Pharmaceuticals 17 (2024): 1470. 39598383 10.3390/ph17111470 PMC11597758

[56] He L, Wang J, Ping F, Association of Glucagon-Like Peptide-1 Receptor Agonist Use With Risk of Gallbladder and Biliary Diseases: A Systematic Review and Meta-analysis of Randomized Clinical Trials. JAMA Intern Med 182 (2022): 513–519. 35344001 10.1001/jamainternmed.2022.0338 PMC8961394

[57] Kim D, Shechtman S, Corinna WS, Use of GLP1 Receptor Agonists in Early Pregnancy and Reproductive Safety: A Multicentre, Observational, Prospective Cohort Study Based on the Databases of Six Teratology Information Services. BMJ Open 14 (2024): e083550. 10.1136/bmjopen-2023-083550 PMC11043712 38663923

[58] Batsis JA, Starr KPN, Villareal Dennis T.. Should the Incretin Hype Be the Same for Older Adults: Promise + Cautions. J Am Geriatr Soc 72 (2024): 2266–2268. 38393783 10.1111/jgs.18816 PMC11226360

[59] Kotecha P, Wenxi H, Yeh YY, Efficacy and Safety of GLP-1 RAs in Children and Adolescents with Obesity or Type 2 Diabetes: A Systematic Review and Meta-Analysis. JAMA Pediatr 179 (2025): 1308–1317. 40952752 10.1001/jamapediatrics.2025.3243 PMC12439189

[60] Kotecha P, Wenxi H, Yeh YY, Efficacy and Safety of GLP-1 RAs in Children and Adolescents with Obesity or Type 2 Diabetes: A Systematic Review and Meta-Analysis. JAMA Pediatr 179 (2025): 1308–1317. 40952752 10.1001/jamapediatrics.2025.3243 PMC12439189

[61] Alemu LS, Sweta N, Jason TA. EBM BLS: Semaglutide Reduces Kidney Disease Progression in Patients with Type 2 Diabetes and Chronic Kidney Disease. J Gen Intern Med (2025): s11606-025-09906-8. 10.1007/s11606-025-09906-8 PMC12960894 41251961

[62] Krisanapan P, Sanpawithayakul K, Pattharanitima P, Safety and Efficacy of GLP-1 Receptor Agonists in Type 2 Diabetes Mellitus with Advanced and End-Stage Kidney Disease: A Systematic Review and Meta-Analysis. Diseases 12 (2024): 14. 38248365 10.3390/diseases12010014 PMC10814593

[63] Lassen MCH, Niklas DJ, Daniel M, Adherence to Glucagon-Like Peptide-1 Receptor Agonist Treatment in Type 2 Diabetes Mellitus: A Nationwide Registry Study. Diabetes Obes Metab 26 (2024): 5239–5250. 39215626 10.1111/dom.15872

